# A Nonparametric SVM-Based REM Recapitulation Assisted by Voluntary Sensing Participants under Smart Contracts on Blockchain

**DOI:** 10.3390/s20123574

**Published:** 2020-06-24

**Authors:** Seung Bum Park, Won Cheol Lee

**Affiliations:** 1 Department of Information and Telecommunication Engineering, Soongsil University, Seoul 06978, Korea; qnatid@gmail.com; 2 School of Electronic Engineering, Soongsil University, Seoul 06978, Korea

**Keywords:** spectrum sharing, radio environment map, Support Vector Machine, Kriging interpolation, blockchain, smart contract

## Abstract

This paper proposes a blockchain-based automated frequency coordination system (BAFCS) for secure and reliable spectrum sharing without causing any harmful interference to an existing system. For the exact assessment of whether the incumbent is interfered with by the spectrum sharer, the received signal strength (RSS) associated with the incumbent should be measured with sufficient accuracy at every location within the area of interest. However, since it requires brute force to carry out empirical measurements around an entire region, to lessen the burden, only the confined portion of the RSSs associated with the incumbent as a kind of primary user are observed and the omitted residuals are conventionally estimated by carrying out the well-known Kriging interpolation with regard to the geostatistical characteristics. This paper proposes a frequency coordination system capable of identifying whether a requested frequency band can be eligible for spectrum sharing while exchanging adequate information over blockchain network to confirm the usability. This paper proposes the Support Vector Machine (SVM)-based Kriging interpolation for recapitulating the radio environment map (REM) when only a fraction of the RSS measurements is acquired by the voluntary sensing participant (VSP). The nonparametric modeling approach for variograms proposed in this paper was determined to have a vital role in making a confident decision regarding spectrum sharing. The simulation result confirmed the effectiveness and the superiority of the proposed BAFCS with several affirmative features, such as enabling the consensus-based approval of spectrum sharing, the secure transaction of the information, and reliable assurance of no harmful interference.

## 1. Introduction

With the prosperity of the fifth generation (5G) era, various types of vertical services, such as autonomous vehicles, drones, and the massive internet of things (IoT), are readily deployed in the real world [1]. In response, due to the rapid growth of the industrial digitalization of private cellular networks [2], the spectrum is becoming more and more congested, and thus frequency resources are becoming too deficient to commercialize a variety of upcoming wireless services [3,4]. To overcome this scarcity of the available spectrum, many carriers and regulation bodies consider spectrum sharing as a felicitous solution. A typical situation where spectrum sharing can be applied is the case where a secondary user is eager to use a certain frequency band. This request can be allowed under the condition of not invoking any harmful interference to the incumbent as a term of use.

Recently, automated frequency coordination was introduced in [5], which can be categorized as a sort of database-driven spectrum management system capable of reliable spectrum sharing [6]. The automated frequency coordination is a system that simplifies existing passive spectrum licensing methods, such as frequency interference effects, feedback collection, and re-examination, and automatically operates the provision of interference protection and available channels in the event of a demand for access from licensed and unlicensed users. Conventionally, there are five operations that need to be deployed as follows:

- Detecting the available spectrum;
- Providing information regarding the validity of spectrum sharing;
- Assessing precise interference impacts to the incumbent;
- Managing the sensing measurement process with involving voluntary sensing participant (VSP);
- Conserving the level of security along the transaction of the information.

In the process of frequency coordination, the prescribed conditions include the secure acquisition of spectrum sensing data and the reliable validation of spectrum sharing. Among these, for the assurance of reliability, it should be mandatory to draw a unanimous consensus from all the incumbents operating on a primary basis. However, it can happen that group of incumbents might refuse to provide their own information regarding the spectrum usage pattern. Thus, it is necessary to shield the information transaction process in secret.

To confirm the validity of spectrum sharing, first, the site-specific received signal strength (RSS) values reached from incumbent transmitters must be observed. Assuming the incumbent is a sort of military weapon system, it is hardly possible to acquire the sensing information without classified authorization for the clearance of security. Thus, some specific sensing information related to the incumbent categorized into the primary user is too delicate to be released; therefore, it should be handled systematically without human intervention. To comply with this requirement, the authors in [7,8] considered spectrum sensing as a service via smart contracts (SPASS), with a spectrum sensing platform based on a smart contract running on blockchain. The researchers in [9] analyzed the feasibility of the application of blockchain deployed for spectrum management with reliable spectrum sharing. This paper deals with two major subjects, the first is the validation process of spectrum sharing running on a blockchain network, and the second is the accomplishment of recapitulating an imperfect radio environment map (REM) [10].

This paper introduces a blockchain platform for the purpose of safe and secure transactions as well as the trust consensus without any leakage of the information to be protected. To perform high confidence decisions on the validity of spectrum sharing, the overall radio frequency (RF) environment surrounding the potential spectrum sharer in the secondary basis should be exploited regarding the pathloss model, diffraction model, antenna height, etc. In addition, a sufficient number of sensing nodes and accurate empirical sensing data (ESD) are required. As the number of sensing nodes increases, naturally, the operating costs and processing time also edge upward. Accordingly, as it is difficult to configure the RF environment perfectly, we promote VSPs by allowing sensing nodes to compensate for the coins in the blockchain. The Kriging interpolation has been adopted in this paper to construct the REM from a certain portion of the ESD. The authors in [11] introduced a method to configure the permissible transmission power of the opportunistic spectrum sharer subject to satisfy the constraint of the outage probability of the incumbent. In this paper, we propose an alternative method to configure an empirical REM applied for the Kriging interpolation with the help of the SVM regression approach.

The blockchain-based automated frequency coordination system (BAFCS) proposed in this paper uses these two essential approaches, essentially running on a blockchain validating spectrum sharing in accordance with assessing the degree of interference with the support of REM recapitulation powered by SVM regression. Thus, at the appearance of the provisional spectrum sharer, the request of spectrum sharing is ignited, all transactions for validating spectrum sharing are automatically executed in order to make the decision of whether the spectrum sharing is approved. The accuracy of REM is critical above everything else [12,13,14,15]. This paper develops a modified Kriging interpolation based on the SVM regression to establish the appropriate variogram model in a nonparametric way rather than a conventional parametric one.

This paper is organized as follows: In Section 2, we introduce the structure and its related operation flow of the proposed BAFCS carrying out the validation of spectrum sharing. In Section 3, we propose a modified version of Kriging interpolation for the recapitulation of REM on the basis of the SVM-based variogram model. Section 4 releases the affirmative features of the proposed BAFCS from the view point of the security and the reliability for spectrum sharing. Specifically, to confirm the confidence of the blockchain operation and how effective the SVM-based Kriging interpolation is, a series of simulations are conducted showing the proper operation of a smart contract and the appropriateness of the interpolated REM. Finally, in Section 5, we present our concluding remarks.

## 2. The Proposed Blockchain-Based Automated Frequency Coordination System (BAFCS)

**Figure 1:**
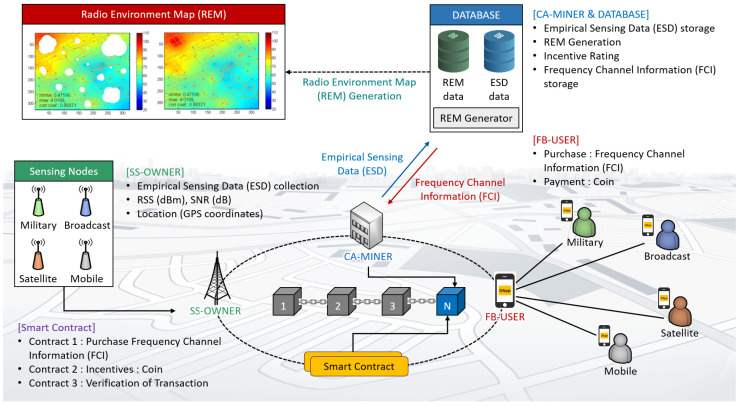
Environmental scenario of the proposed blockchain-based automated frequency coordination system (BAFCS) operation.

In this section, in regard to the secure transactions of information encompassing the status of spectrum utilization related to the incumbent, the empirical sensing data, etc., the overall functions composing the proposed BAFCS are introduced. The proposed BAFCS pursues verifying whether the spectrum sharing can be approved when requested by the secondary user as in Figure 1, in which the incumbent is in service. Here, the proposed BAFCS is based on blockchain theory [16,17], which enables secure and trusted transactions of highly confidential information [18].

The scenario of the proposed BAFCS shown in Figure 1 is the version that employs only one CA-MINER. The more complete structure for BAFCS is to build a blockchain system comprising multiple CA-MINERs in a distributed fashion. Thus, when enlarging the service region and aggravating competition for the reduction of cost, the more CA-MINERs would appear as Certification Agencies. As a result, a more commercially distributed blockchain infrastructure can be constructed for realistic BAFCS.

Along the operation of BAFCS, the REM configured by ESD is the basic raw material, as the constructed REM enables assessment of the impact of the interference to the incumbent. To grant the permission for the accessibility of the shared band, it is mandatory to acquire the unanimous consensus among radio service providers (RSPs) working on a primary basis. For this, it is necessary to observe the RSS associated with the RSP required to be protected from the unwanted interference radiated by the spectrum sharer. This paper considers several groups of VSPs, namely the delegation of individual RSPs, whose role is the collection of RSSs and belongs to its own RSP. At the completion of their work, the VSP is paid by coins in a form of cryptocurrency as the reward for the sensing labor. There are five key features in the proposed BAFCS as follows:

- Collecting ESD from the domain-delegated VSPs to configure the REM in the region of interest;
- Recapitulating the site-specific REM with the assistance of the Kriging interpolation;
- Rewarding the coin to the VSPs with the comprehensive assessment;
- Transacting the information and exchanging the cryptocurrency as verified by smart contracts; and
- Deciding whether the request for the spectrum sharing is approved.

In the proposed of BAFCS, the smart contract running on blockchain is essential to verify various transactions subject to fulfilling a series of prescribed requirements. A smart contract can be designated as a programming code confirming the validation of the transactions purchasing the ESD and paying the VSP in a form of cryptocurrency [19,20]. The reliability and secureness in the information transaction, the coin remittance and the collection and decision for the possibility of spectrum sharing should be investigated. All the RSPs have their own roles and responsibilities, which are checked and assessed by conducting appropriate smart contracts in the blockchain network. The definitions and roles of entities participating in the proposed BAFCS are as follows:

- Certification Agency (CA-MINER)
  The CA-MINER takes charge of the REM configuration upon the aggregation of the ESD delivered from the SS-OWNER, and verifies the mining and all types of transactions. As the labor cost of the verification of transactions and the REM and ESD management, coins will be paid as a reward. The CA-MINER may be designated as the trusted governmental agency, it could be possible to handle the sophisticated spectrum utilization information. Here, since the blockchain platform manages all transactions related to the data exchange, it can maintain a secure treatment of highly classified information.
- Sensing data Sellers (SS-OWNER)
  The SS-OWNER collects the sensing information on behalf of its own RSP by sparsely distributed VSPs equipped with sensing devices under a certain terms of the contracts with each RSP. The SS-OWNER sends the aggregated ESD to the CA-MINER, and then the SS-OWNER pays the VSPs in the form of coins under a smart contract. Further, ESD can be utilized for analyzing the impact of interference emitted from the spectrum sharer categorized for the purpose of local wireless services. The information related to ESDs could be RSS, signal-to-interference plus noise ratio (SINR), or geolocation relevant to the VSP position. The amount of coin rewarded to the VSP is dependent on how accurate the ESD is, and how frequent the data they have collected are.
- Frequency use Buyers (FB-USER)
  The FB-USER is regarded as an opportunistic spectrum sharer, who would like to buy the information regarding the validity of spectrum sharing. These FB-USERs can be new 5G private networks for vertical business entrances or local RSPs. They are likely to utilize the shared band for the purpose of launching new wireless services on a spectrum sharing basis. At the initial stage, an FB-USER begins to request the permission to CA-MINER by paying the certification processing charge as a deposit. Once the permission is approved, the overall transaction process is completed and then the payoff transaction is followed to give the incentive to the VSPs.

**Figure 2:**
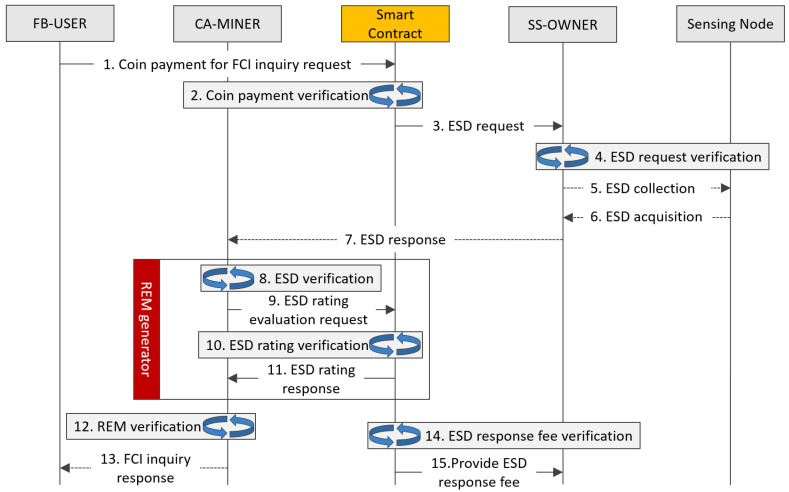
Overall operation process between entities composing the BAFCS.

The FB-USER, SS-OWNER, and CA-MINER deliberately exchange the data and signals relevant to the request and response. For each transaction routing, smart contracts begin to run whenever a FB-USER ignites the request of spectrum sharing as shown in Figure 2. The FB-USER purchases the critical information reflecting the validity of spectrum sharing along the prescribed frequency band and pays a certain amount of coins for further verification. Once the smart contract runs, a certain amount of coins as the certification processing charge is remitted from FB-USER, and subsequently saved in an intermediate account as a security deposit on the smart contract.

Then, the SS-OWNER begins to aggregate the sensing information measured from sparsely distributed VSPs in the region of interest. At the completion of the sensing and collection process, the aggregated ESD is delivered subsequently to CA-MINER for the assessment of the quality of ESD by grading the degree of accuracy and normality to determine the incentive ratings prescribed by the smart contract. Furthermore, CA-MINER builds up the REM to analyze the impact of the interference arising from the FB-USER and whether it is within tolerable levels. Once the validity of the spectrum sharing is confirmed upon the consensus process, the FB-USER is informed that they have permission for spectrum sharing and the SS-OWNER remits this to the VSPs.

## 3. Recapitulation of Site-Specific REM Using SVM-Based Kriging Interpolation

This section introduces the modified Kriging interpolation via adopting SVM regression for modeling the trend of variograms in a nonparametric fashion, which yields the REM reflecting a geostatistical characteristic [21,22]. At the initial stage, due to the insufficiency of measurements, many points are omitted in REM constructed by empirical ESDs. To fill up the REM, the geostatistical analysis should be explored in advance, in order to perform the Kriging interpolation. The trend of spatial covariances can bring out the variogram as a spatially lagged second-order moment. After the mathematical exploitation of these, the resulting variograms could be fitted to an approximate model, and then the trend of the variogram can be fitted to one of the well-known template functions. This is a conventional way of configuring a variogram in parametric modeling. However, in this paper, in light of the SVM-based regression, this section introduces a nonparametric modeling approach in replacement of the conventional approach. After the fitting of the variogram in the regression manner, the resulting model dictates the relationship between the spatial distance lag and the spatial covariance, indicating the degree of similarity. Considering the variogram, since the second-order statistics in the spatial domain is assumed to be stationary, the site-specific variogram only depends on the spatial lag interval in the region of interest. This is well-described as the quasi-stationarity mentioned in [23].

**Figure 3:**
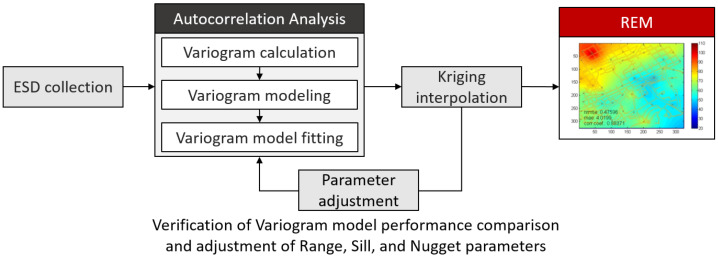
Block diagram of the Kriging interpolation process for the REM.

Figure 3 shows the flow of recapitulating the incomplete REM with the help of the Kriging interpolation provided that some portion of ESD is given a priori. Here, as mentioned before, the sparsely distributed VSP engaged in each RSP carry out the measuring task. Conventionally, the variogram modeling is accomplished by selecting the most suitable template among the well-defined mathematical functions. The criterion for choosing an appropriate one is inspecting how similar the modeled variogram is to that calculated from the given RSSs. Then, if the best fit of the variogram is exploited, it is ready to conduct the Kriging interpolation to configure the REM in the region of interest at the current time.

### 3.1. Variogram Exploitation with Given Site-Specific Sensing Measurements

The variogram represents the characteristics of spatial covariance between two spatially apart measurements. Thus, it can state that the variogram only alter its value depending not on the geolocation but the spatial lag [23]. Once the model of the variogram is determined, the spatial covariance can be evaluated by varying the distance of the two measurement locations. Provided that the location of the measurement point and the RSSs are known a priori, the variance values γ(d) are calculated by the following Equation (1):

(1) $$\gamma (d)=\frac{1}{2N_{d}}\sum_{i,j}{\left(Z_{\left(s_{i}\right)}-Z_{\left(s_{j}\right)}\right)}^{2}$$

where *d* is the distance between two locations denoted by $s_{i}$ and $s_{j}$ and $N_{d}$ denotes the number of all ESD points used. In addition, $Z_{\left(s_{i}\right)}$ is the measured ESD at $s_{i}$, and $Z_{\left(s_{j}\right)}$ is another ESD measured at $s_{j}$ separated by a certain distance. It is natural that, as the distance increases, the variance enlarges, conversely, the variance becomes small as the distance decreases. Generally speaking, the variogram model is featured by three major parameters, which are the Range, the Nugget, and the Sill. Here, the Range denotes the maximum distance in which we can say that two measured ESDs are correlated, and the Sill is the variogram value corresponding to the Range. Next, the Nugget denotes the variance value at zero distance. If either the two points are close or the relevant scattering distribution is dense, the spatial covariance between any two points naturally increases, so that it is easy to select appropriate parameters. After selecting the Range, Nuget, and Sill parameters, the variogram modeling can be configured conventionally by choosing an appropriate template model among four popular variogram models, such as the linear model, spherical model, exponential model, and Gaussian model [23]. The accuracy of the Kriging interpolation is directly related to how well the estimated variogram reflects the RF environment. In the conventional approach, to point out the appropriate variogram model, it is a prerequisite to select the most plausible parameters, namely Range, Sill, and Nugget [24].

Once the variogram model is fixed, then the weights for the interpolation could be obtained in the minimum mean squared error (MMSE) sense. Concisely speaking, the Kriging interpolation is carried out in a conventional way by performing a simple linear combination as in Equation (2) with the given ESD measured $s_{1}$, $s_{2}$, ..., $s_{n}$, together with calculated weights as follows:

(2) $$Z_{\left(s_{0}\right)}=\sum_{i=1}^{n}\lambda_{i}Z_{\left(s_{i}\right)}$$

where $s_{0}$ is the location at which ESD is missing, $Z_{\left(s_{0}\right)}$ denotes the interpolated value at $s_{0}$, and $Z_{\left(s_{i}\right)}$ denotes the measured ESD at the site $\left(s_{i}\right)$ where there is a VSP carrying out their sensing duty. Specifically, *n* denotes the total number of available empirical measurements that are utilized to exploit the variogram model as well as perform the Kriging interpolation, and $\lambda_{i}$ means the value for weighting ith ESD. As mentioned in [23], there are three types of Kriging interpolation, which are classified in terms of the feature of the spatial correlation characteristic. In this paper, the ordinary Kriging interpolation is employed provided that there are weights subject to obeying the non-bias condition as shown in Equation (3) as follows:

(3) $$\begin{array}{cccc} \; & \underset{\gamma ,\lambda }{\mathrm{minimize}}\; & \; & \gamma_{\left(s_{0}\right)}-2\sum_{i=1}^{n}\lambda_{i}\gamma_{\left(s_{0},s_{i}\right)}+\sum_{i=1}^{n}\sum_{j=1}^{n}\lambda_{i}\lambda_{j}\gamma_{\left(s_{i},s_{j}\right)}\\ \; & \mathrm{subject}\;\mathrm{to}\; & \; & 1-\sum_{i=1}^{n}\lambda_{i}=0 \end{array}$$

where $\gamma_{\left(s_{0}\right)}$ denotes the variance of the estimation error induced by deploying the ordinary Kriging interpolation, and $\gamma_{\left(s_{i},s_{j}\right)}$ indicates the spatial covariance between distinct ESDs at the two different sites $s_{i}$ and $s_{j}$. The ordinary Kriging interpolation is used to calculate the weights for all points subject to minimizing the error variance while obeying the constraint that the sum of all weights is the unity [21]. Additionally, the ordinary Kriging interpolation can be expressed in a matrix, such as Equation (4), and a matrix with the size of (n+1)×(n+1) is generated as

(4) $$\left[\begin{array}{ccccc} \gamma_{\left(s_{1},s_{1}\right)} & \gamma_{\left(s_{1},s_{2}\right)} & \cdots & \gamma_{\left(s_{1},s_{n}\right)} & 1\\ \gamma_{\left(s_{2},s_{1}\right)} & \gamma_{\left(s_{2},s_{2}\right)} & \cdots & \gamma_{\left(s_{2},s_{n}\right)} & 1\\ \vdots & \vdots & \ddots & \vdots \\ \gamma_{\left(s_{n},s_{1}\right)} & \gamma_{\left(s_{n},s_{2}\right)} & \cdots & \gamma_{\left(s_{n},s_{n}\right)} & 1\\ 1 & 1 & \cdots & 1 & 0 \end{array}\right]\left[\begin{array}{c} \lambda_{1}\\ \lambda_{2}\\ \vdots \\ \lambda_{n}\\ 0 \end{array}\right]=\left[\begin{array}{c} \gamma_{\left(s_{0},s_{1}\right)}\\ \gamma_{\left(s_{0},s_{2}\right)}\\ \vdots \\ \gamma_{\left(s_{0},s_{n}\right)}\\ 1 \end{array}\right].$$

### 3.2. SVM-Based Kriging Interpolation Model

**Figure 4:**
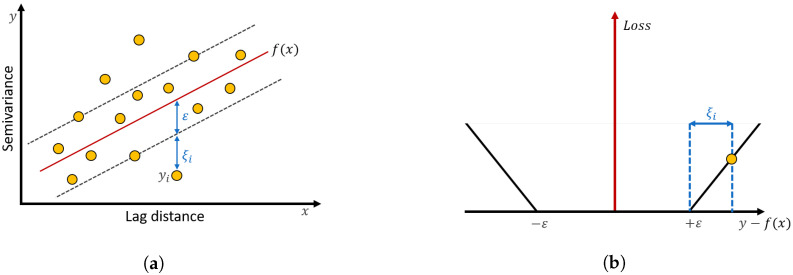
(**a**) The SVM regression. (**b**) The *ε*-insensitive loss function.

As described earlier, the variogram model used for the Kriging interpolation is characterized in terms of three major parameters, Range, Sill, and Nugget. In general, these parameters are achieved by performing an exhaustive search until the configured curve fits the trend of the calculated semivariance values from the measured ESDs. This trial and error approach is so inefficient that the computational burden becomes enormous. To circumvent this difficulty, this paper employed the SVM regression to assist the configuration of the variogram model autonomous time-consuming exploitation [25,26]. For the classification of the data set, it is salient to partition off the data into several groups with a sufficient separation margin to ease the classification. The objective of SVM regression exploits the median curve among data at a certain value on the abscissa, which reflects the geostatistical characteristics as shown in Figure 4a. If the regression curve is configured in a linear function, this can be expressed by $f\left(x\right)=w^{T}x+b$, where *x* denotes a lag distance value representing the distance between two measurement points, *w* denotes a weight, *b* denotes a parameter determining the shift of the regression line. In order to achieve the regression line f(x), it is necessary to find the margin specifying the degree of allowance for the existence of the data set, in which the loss corresponding to the data within the allowance range is treated as zero, as shown in Figure 4a.

This intentional margin can prohibit the regression curve being overfitted. At last, the mentioned ε-insensitive loss function $L_{\varepsilon }$ can be expressed as in Equation (5):

(5) $$L_{\varepsilon }\left(y_{i},f(x_{i})\right)=\{\begin{array}{cc} 0, & |y_{i}-f(x_{i})|\;\leq \;\varepsilon +\zeta_{i}\\ |y_{i}-f(x_{i})|-\varepsilon +\zeta_{i}, & \mathrm{otherwise} \end{array}$$

where ε denotes the margin for SVM regression, $y_{i}$ denotes the semivariance value associated with lag distance $x_{i}$, and $\zeta_{i}$ denotes a slack variable for assigning the penalty, as shown in Figure 4b, to enhance the appropriateness of the regression line f(x) to compromise either the overfitting or the underfitting. Next, the following optimization subject obeying the constraints gives rise to the regression line, which supports the Kriging interpolation for the unknown RSS value by applying the ε-insensitive loss function as in Equation (6): for all i=1,⋯,m

(6) $$\begin{array}{cc} \underset{w,b,\zeta_{i},\zeta_{i}^{*}}{\mathrm{minimize}}\; & \frac{1}{2}{\left||w|\right|}^{2}+C\sum_{i=1}^{m}\left(\zeta_{i}+\zeta_{i}^{*}\right)\\ \mathrm{subject}\;\mathrm{to}\; & y_{i}-\left(w^{T}x_{i}+b\right)\;\leq \;\varepsilon +\zeta_{i}\\ \; & \left(w^{T}x_{i}+b\right)-y_{i}\;\leq \;\varepsilon +\zeta_{i}^{*}\\ \; & \zeta_{i},\zeta_{i}^{*}\;\geq \;0 \end{array}$$

where ||w|| denotes the size of the margin, and *C* denotes a penalty value that determines the magnitude of the error tolerance by $\zeta_{i}$, $\zeta_{i}^{*}$. For the ESD points, if $y_{i}$ is inside of the ε-tude, the error $y_{i}-f(x_{i})$ is less than ε, and it does not require a non-zero slack variable $\zeta_{i}$, $\zeta_{i}^{*}$, equivalently speaking, then, the slack variable $\zeta_{i}$, $\zeta_{i}^{*}$ could be set to zero. Thus, the purpose of applying SVM regression is to minimize the size of the error tolerance, provided that all the semivariances are included inside the range of error tolerance [27]. Next, the Lagrangian multiplier and Karush–Kuhn–Tucker (KKT) conditions are used to calculate the regression line, as shown in Equation (7) as follows:

(7) $$\begin{array}{c} f(x,a_{i},a_{i}{}^{*})=\sum_{i=1}^{n}\left(a_{i}-a_{i}{}^{*}\right)K\left(x,x_{i}\right)+b \end{array}$$

where $a_{i}$, ${a^{*}}_{i}$ are the Lagrange multipliers, and *x*, $x_{i}$ denotes the position coordinate [28]. $K(x_{i},x)$ denotes the kernel function. The kernel function maps a certain low-dimensional data point into a high-dimensional one, easing either the classification or the regression in a nonlinear form [29]. In this paper, the semivariance value and lag distance value, which are input parameters for calculating the SVM regression, are in the form of nonlinearity. Therefore, the kernel function must be used, the popular kernel functions that are frequently and widely used are the linear kernel, radial basis function (RBF) kernel, and polynomial kernel. In this paper, the RBF kernel, in favor of a relatively fast computation speed, is applied for nonlinear classification expressed in Equation (8) as follows:

(8) $$K\left(x,x_{i}\right)=e^{-\sigma |x-x_{i}{|}^{2}}$$

where σ denotes the standard deviation value of the Gaussian method. By employing the RBF function for modeling the variogram, it can be possible to trace the trend of spatial covariance values evaluated by distance lag in a nonlinear fashion. Applying the SVM regression described earlier, it is an affirmative feature that nonparametric variogram modeling is possible to implement without considering the determination of parameters, such as the Range, Nugget, and Sill parameters. Thus, due to the absence of an exact mathematical formula for the variogram model, the Kriging interpolation should be conducted with regard to the regression line rather than using a prescribed relationship [30,31].

## 4. Simulation

To verify the plausibility of the proposed BAFCS, the relevant simulation was performed to ensure blockchain functionalities as well as SVM-based REM recapitulation. Upon the request from the opportunistic spectrum sharer, the proposed BAFCS attempts to validate whether the spectrum sharing can be approved through drawing consensus unanimously and automatically without any human intervention together with the acquisition of empirical sensing data from VSP to be rewarded. Truly speaking, this section consists of two parts, the first part provides the assurance of the operation of the blockchain platform making sure that every smart contract works properly from the points of view of the request, the verification, and the response. The second part deals with the proposed SVM-based variogram modeling, which is further used for the Kriging interpolation to recapitulate the incomplete REM.

### 4.1. Assurance of the Functional Operations on the Blockchain Platform of the Proposed BAFCS

The blockchain platform running on the proposed BAFCS is composed of three major functionalities called entities, which are namely the SS-OWNER, FB-USER, and CA-MINER.

**Figure 5:**
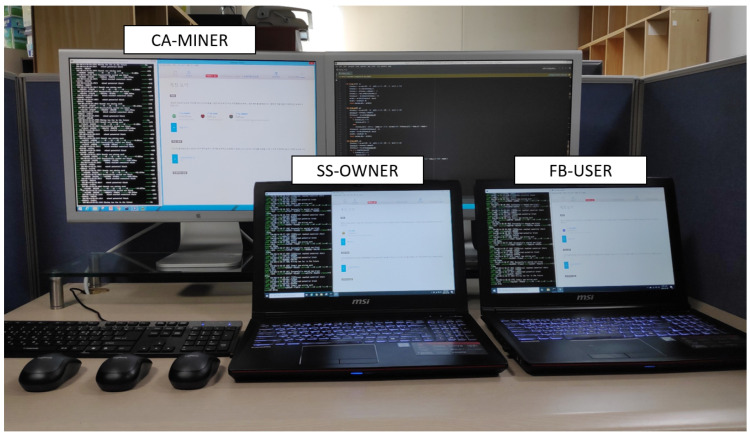
The experimental environment for operating blockchain-based transactions on BAFCS.

In order to conduct the computer simulations ensuring the appropriateness in the operations, the simplified simulation environment for the blockchain platform is constructed, where three notebooks are designated entities for the functioning of CA-MINER, SS-OWNER, and FB-USER, respectively, as shown in Figure 5.

In accordance with conducting a realistic simulation, commercial LTE mobile phones, described as VSPs, were used to play the role of collecting the ESD. Details regarding the roles of the FB-USER, SS-OWNER, and CA-MINER are shown in Figure 1, and the blockchain network is intended to be working on the basis of private Ethereum programmable by conducting Go-Ethereum with operations that are checked and monitored by the Mist-Browser. The Mist-Browser is used as a tool to monitor the programming of smart contracts, transactions between users, and the operation of the Ethereum blockchain network. In the course of performing consecutive transactions, basic operations can be categorized into several pairwise interactive transactions, such as the request, the response, the deposit, and the payment, etc. Every transactions must be verified by smart contract, whose roles and responsibilities are summarized in Table 1.

**Figure 6:**
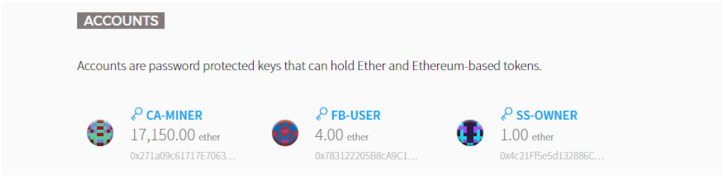
The results before performing the smart contract in Mist-Browser.

For the sake of convenience, the transaction operation is verified entity-by-entity running on BAFCS, the FB-USER and SS-OWNER respectively. In the course of transaction with each other, CA-MINER entity has the exact same role and responsibility as a miner as the key entity composing the blockchain network. Figure 6 shows the exposed status of the initial stage for transaction via capturing Mist-Browser, where it can notice four ETH in the FB-USER’s account that is the amount of the fee for the verification indicating the usability of the shared spectrum per request basis. There is also the amount of one ETH in the SS-OWNER’s account. Further, after the aggregation and the delivery of sensing data to the CA-MINER acquired from the VSPs, the appropriate charge is deposited into that account and remitted by the corresponding smart contract.

The BAFCS begins to operate with the function of pay_coin() on a smart contract purchasing the validation analysis at a specific site where a FB-USER expects to use a specific frequency band on a spectrum sharing basis. For purchasing the confirmation of validation, the FB-USER remits 1 ETH to the smart contract as a security deposit.

**Figure 7:**
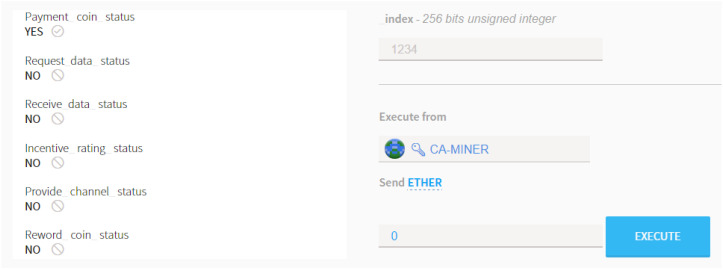
The status change at the start of a smart contract in Mist-Browser.

After the payment action, the smart contract recognizes the deposit and changes its status to YES as shown in Figure 7. After the function of pay_coin() is complete, the SS-OWNER is requested to aggregate the ESD via executing the function of tx_data().

**Figure 8:**
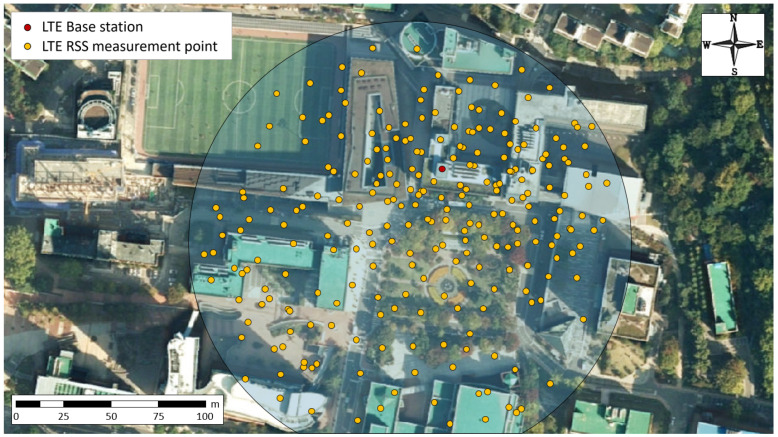
The RSS points in the Soongsil University campus environment.

The SS-OWNER collects the ESD captured by sparsely distributed VSPs equipped with LTE mobile phones. To gather the sensing data, in our experiment, the ESD were measured realistically at the campus of Soongsil University by sparsely distributed VSPs equipped with LTE mobile phones, as shown in Figure 8. After the overall collection process, by activating rx_data(), the aggregated ESD are delivered to the CA-MINER automatically, and then stored in the database. After the accomplishment of the sensing process, the VSPs that provided sensing labor are rewarded in sequence upon the grading of VSPs through inspection of the accuracy and precision. Assessing the quality of the sensed data that belongs to each VSP is executed in sequence via performing ch_grade().

Concisely speaking, ch_grade() is executed for the validity of the spectrum sharing with regard to the guidelines in [32]. Accordingly, the LTE RF condition can be categorized into four classes upon the observation of RSS. In this paper, it was developed to compensate with coins if the RF conditions met or exceeded the Good condition in accordance for the validity of the spectrum sharing. Here, whether the ESD were satisfactory or not, the incentive as the recompense for laboring voluntary sensing was assigned distinctively and paid to the VSPs. Thereafter, upon the aggregated ESD interference analysis would be performed via analyzing the impact of a FB-USER provided that the RF environment was understood.

In our simulation, only the pathloss model was applied to determine whether the impact was serious. Here, the validity of the spectrum sharing can be approved by inspecting the calculated SINR, provided that the transmission specifications of the FB-USER are prescribed a priori given its geolocation. Then, the value of the SINR at a certain location can be calculated and compared with a prerequisite limitation to be complied in sequence. Therefore, if the calculated SINR is less than the required criterion, then FB-USER can not apply the spectrum sharing as a matter of course. In this situation, the only way for being validated for spectrum sharing is to decrease the transmit power at the abandonment of service coverage.

**Figure 9:**
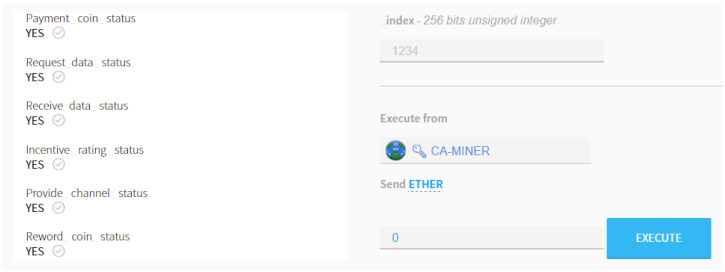
Status changes after smart contract completes in Mist-Browser.

At the completion of the incentive rating process, the Kriging interpolation begins to recapitulate the REM around the area of interest. Upon the result of the recapitulated REM, the interference analysis is performed consecutively by observing the SINR status. Then, the determination can be made regarding whether the spectrum sharing can be performed. Finally, the coin as the cryptocurrency previously stored in the account of the smart contract is withdrawn to remit coins to the SS-OWNER via executing the command of rew_coin(). Figure 9 shows the captured screen of a Mist-Browser showing the result of the operation conducted by smart contracts.

At the instant that the overall routine for verification is over, at the sequel, all the executions accomplished by a group of smart contracts are completed, and as shown in Figure 9, all the statuses are automatically changed from ’NO’ to ’YES’. This sequential alteration indicates that the smart contract is working properly without any misoperation. Then, the new block is generated and attached to the existed blockchain by CA-MINER, which is the completion sign of the secured verification process reading that all the transactions were properly executed.

**Figure 10:**
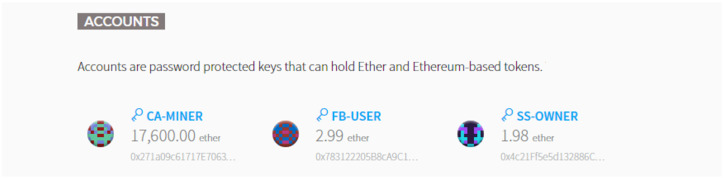
Results after operating the smart contract in Mist-Browser.

In addition, CA-MINER receives the recompense for mining labor as shown in Figure 10. Clearly, the balance of FB-USER is reduced to 2.99 ETH and the balance of SS-OWNER is increased to 1.98 ETH. This is due to the withdraw and deposit actions approved by the smart contract function. The whole process in the proposed BAFCS is done automatically without any human intervention, along the collection of the ESD, the secure verification upon the unanimous consensus, and the coin transactions. There is a specific drawback on the employment of the smart contract that is attributed to the increase of cost as the length of the code is longer. If more users associate with a certain blockchain network, a more complicated class of security is necessary. It is left to be resolved how to reduce the charge of using smart contracts while sustaining the user accessibility.

### 4.2. Performance Analysis of the Proposed REM Recapitulation

This section exploits the plausibility of the proposed REM recapitulation scheme provided that only a limited portion of the ESD is known. To fill the omitted measurements around the region of interest, the Kriging interpolation is adopted in this paper with the employment of SVM regression to configure a plausible quality of variogram model. Upon considering the propagation behavior, there may be many clutters that disturb the propagation depending on the RF environment. Reflecting the geographical environment, the levels of RSS values spread over this are measured and treated as the ESD. In the simulation, a number of ESD were collected at the locations in Soongsil University campus in Figure 8. In addition, the development of the Kriging interpolation was done in Python, and the REM recapitulation was executed by the Kriging interpolation with the help of the SVM regression allowing for configuration of the geostatic variogram model, in which the kernel function is implemented by the RBF function developed with the sklearn package of Python.

**Figure 11:**
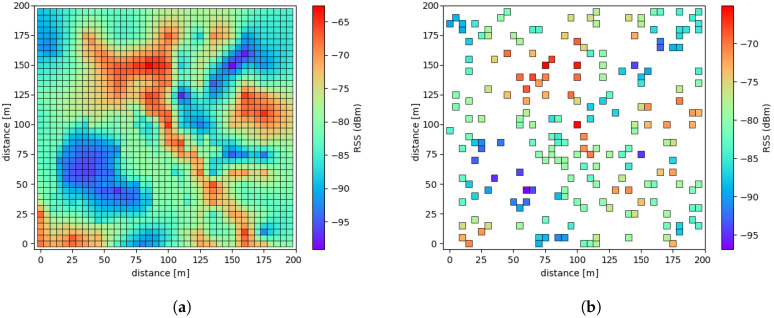
REM with (**a**) 1600 pixels, i.e., the original empirical sensing data (OESD) and (**b**) 200 pixels randomly chosen.

The area of interest is shown in Figure 11a, in which the area is the main campus district of size 200 m by 200 m. This district is partitioned purposely into 1600 pixels units, where each pixel is a square with the size of 5 m by 5 m. Although the sensing should be conducted at every 1600 points to acquire the perfect REM, this was too difficult to acquire the entire ESD exhaustively. For the sake of convenience, we measured 320 sparsely distributed ESD only, which were purely ESDs around the area of interest. Due to the original REM being composed of 1600 ESD to be compared with the results from conducting the proposed method as well as the other conventional approaches, we artificially extrapolated from the empirically obtained REM with only 320 ESD into the REM with 1600 ESD through performing a general-purpose image extension scheme.

Hereafter, the value at each pixel is treated as the original empirical sensing data (OESD) for further performance comparative works. It is possible to make the incomplete version of REM by conducting random subsampling on pixels in REM having 1600 OESD. For example, a REM with 200 OESD can be constructed by selecting random pixels from a REM with 1600 OESD as in the original in simulation even though it is artificially generated as shown in Figure 11b. Here, RSS values treated as the ESD can be distinguished between red and blue areas, in which red represents a good signal area, whereas, blue indicates a shading area.

**Figure 12:**
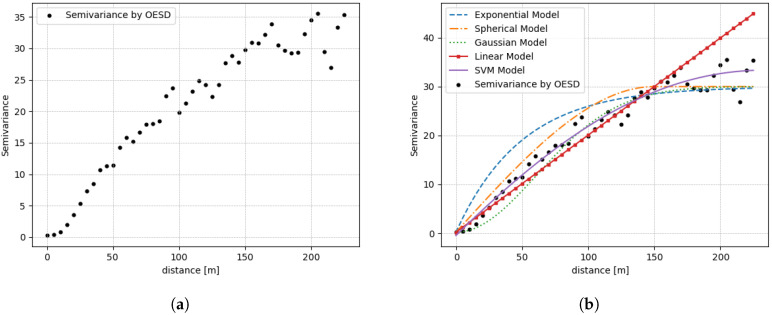
(**a**) Semivariance trends calculated by OESD. (**b**) Various trends of the fitted variogram model

Figure 12a shows the semivariance calculated with regard to [23] from the original REM composed of 1600 ESD as the distance lag increased. Clearly, the trend of the semivariance was not weak; however, the variogram model was needed for further Kriging interpolation. Conventionally, variogram trends were fitted by the parametric model described in many articles, which has been popularly utilized and generally represented by several template functions parametrized in terms of Range, Nugget, and Sill. However, due to the difficulty of determining these parameters, this paper proposed SVM-based variogram modeling, which can be designated as a nonparametric approach characterized as purely data-driven modeling without any consideration of the manual adjustment as mentioned before.

Figure 12b shows the various types of fitted variograms following the trend of semivariance distribution with four parametric models and the proposed SVM-based nonparametric model. Here, the major difference between the parametric model and the nonparametric one is originated whether prior information about Range, Nugget, and Sill is given or not. To construct the parametric variogram model, the Range, Nugget, and Sill values are elaborately set to 28, 0.3, and 30, respectively. The SVM-based approach generated a variogram model that does need not these parameters but instead the semivariances computed from the ESD. To enhance the accuracy of the SVM-based variogram model, it was preferable to acquire a moderate amount of data without having any far erroneous sensing data. To assess the similarity between the original REM with a full 1600 OESD and the recapitulated version of REM obtained from a given limited number of OESDs less that 1600, the root mean square error (RMSE), as well as the mean absolute percentage error (MAPE), were evaluated. Here, the incomplete version of REM was obtained by conducting random subsampling on the original REM with 1600 OESD.

Table 2 presents the results. The similarity resulting from applying the proposed SVM-based veriogram model was superior to the conventional approaches adopting parametric variogram modeling, quantitatively. The COST 231 Hata model was used to compare the performance of the variogram models, and the parameter values were set with the center frequency at 2630 MHz, antenna gain at 0 dbi, antenna height at 1.5 m, and transmitter power at 23 dBm [33,34]. The COST 231 Hata model is one of the pathloss models used in LTE environments, and the performance of the RMSE and MAPE values was reduced compared to variogram models as it can only see attenuation trends in the radio environment without identifying shaded areas, such as the terrain or buildings.

**Figure 13:**
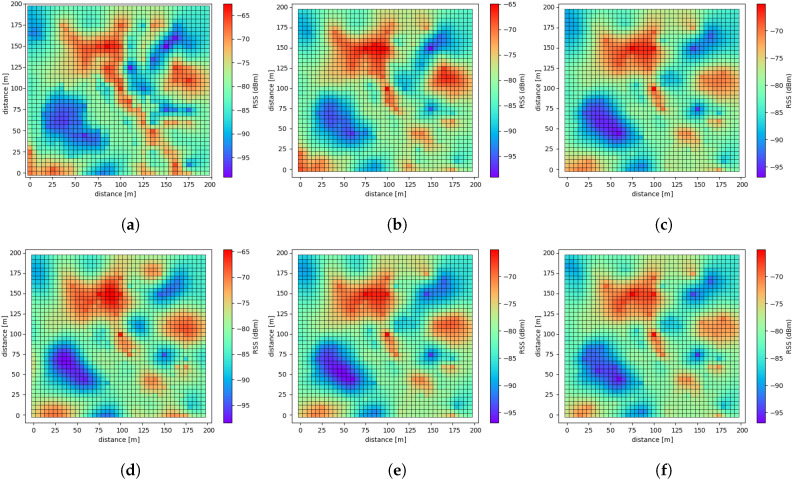
(**a**) Original REM constructed by OESD. (**b**) REM based on the proposed SVM regression model. (**c**) REM based on the linear model. (**d**) REM based on the Gaussian model. (**e**) REM based on the spherical model. (**f**) REM based on the exponential model.

The Kriging interpolation was conducted regarding the selected modeling approach, resulting in recapitulated REMs as shown in Figure 13. Here, to discriminate the performance difference, we considered the situation in which only 200 OESDs were available, randomly chosen from the full version of OESD, and the rest were estimated by executing the Kriging interpolation. Figure 13 shows that different kinds of recapitulated REMs were configured distinctively on the basis of variogram modeling. Table 2 concisely indicates how similar the original REM and the recapitulated are quantitatively in the sense of either the RMSE or MAPE.

**Figure 14:**
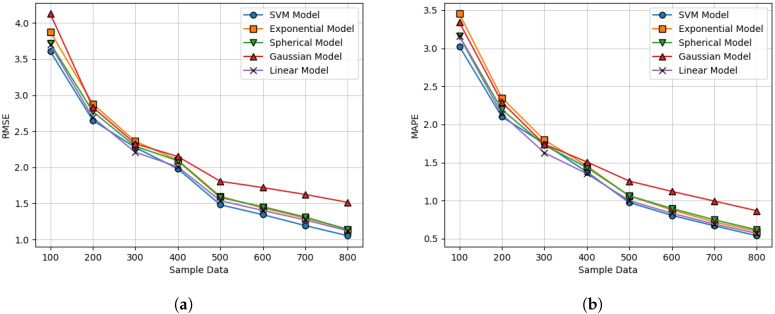
Performance comparison for the distinctive variogram modeling approach according to the number of ESDs in the sense of minimizing (**a**) the RMSE, and (**b**) the MAPE.

According to Table 2, the proposed SVM-based variogram modeling approach holds the superior performances in both the RMSE and MAPE. In Figure 13, the REM based on the SVM-based approach looks similar at a glance. If the more number of OESD were utilized, the performance gap became negligible. To obtain more insightful results, we observed the RMSE and the MAPE increasing the number of OESD from 100 to 800 in steps of 100. Figure 14 shows the corresponding RMSE and MAPE results, and we concluded that the proposed SVM-based model ensured a promising performance. Here, the cases above 800 OESD are omitted because the relevant comparison became meaningless.

## 5. Conclusions

In this paper, we proposed a blockchain-based automated frequency coordination system (abbreviated as BAFCS) that realized secure and trusted spectrum sharing upon a unanimous consensus basis without any intentional human intervention. The major role and responsibility of the proposed BAFCS was the confirmation of validity of spectrum sharing for carrying out secure transactions without leakage or disclosure of the classified information. On determining whether a specific frequency band could be utilized in a spectrum sharing basis under the grant of approval by the terms of use, it was then essential to assess the expected impact of the interference beforehand for the receiver of the incumbent signal emitted from the FB-USER, who was designated as the provisional spectrum sharer.

Toward this, finely measured RSS values, namely ESD comprising the REM, needed to be fully acquired within the area of interest. However, as it required brute force to collect a tremendous number of ESDs, this paper proposed a modified version of Kriging interpolation that was capable of enhancing the resolution of incomplete REMs via adopting the SVM regression method applied for a variogram model in a nonparametric fashion. The affirmative features attributed to the SVM regression had no need to specify parameters, such as Nuget, Range, and Sill, and the more plausible determination of the variogram model produced the recapitulated REM precisely. By conducting the computer simulation, the entities comprising the proposed BAFCS were inspected by checking if all the functionalities together with the verification operations of smart contracts were carried out appropriately. In conclusion, the proposed BAFCS is a promising methodology for the future regime of spectrum sharing for conducting reliable and trusted validation with the assistance of VSPs. 

## Figures and Tables

**Table 1:** Function types and functions of a smart contract.

Function Name	Smart Contract Role
pay_coin()	1 Ethereum (ETH) payment when purchasing FCI
tx_data()	Request ESD from SS-OWNER
rx_data()	Providing the collected ESD
ch_grade()	Assigning coins by incentive grade
ch_data()	Providing the FCI to FB-USER
rew_coin()	Providing the ESD response fee 1 ETH to SS-OWNER

**Table 2:** Minimum root mean square error (RMSE) and mean absolute percentage error (MAPE) values for each variogram model and the COST 231 Hata model.

Model	SVM	Exponential	Spherical	Gaussian	Linear	COST 231 Hata
RMSE	1.81	4.46	2.94	2.61	4.58	7.42
MAPE	1.23	4.11	1.44	1.32	2.90	6.71
